# H&E-based MSI/MMR testing with AI in colorectal cancer: a multi-centred blinded evaluation

**DOI:** 10.1038/s41746-025-02218-5

**Published:** 2025-12-15

**Authors:** Cher Bass, Foivos Ntelemis, Julian Schmidt, Steffen Wolf, André Geraldes, Debapriya Mehrotra, Shikha Singhal, Narender Kumar, Angelica Marcia, Nicholas Bennett, Oscar Maiques, Mitchell Hyde, Bejal Mistry, Grace Rogerson, Michele Cummings, Clare Freer, Elizabeth Walsh, Manuel Salto-Tellez, Maurice Loughrey, In Hwa Um, David J. Harrison, Richard Clarkson, James Blackwood, J. Carl Barrett, Jakob Nikolas Kather, Nicolas M. Orsi, Pahini Pandya, Salim Arslan

**Affiliations:** 1 Panakeia Technologies, Salisbury House, Station Road, CB1 2LA Cambridge, United Kingdom; 2 Women’s Health Research Group, Leeds Institute of Cancer & Pathology, Wellcome Trust Brenner Building, St James’s University Hospital, University of Leeds, Beckett Street, Leeds, LS9 7TF United Kingdom; 3 Barts Cancer Institute, Queen Mary University of London, Charterhouse Square, London, EC1M 6BQ United Kingdom; 4 Department of Histopathology, St James’s University Hospital, Leeds Teaching Hospitals NHS Trust, Leeds, United Kingdom; 5 Queen’s University Belfast, School of Medicine, Dentistry and Biomedical Sciences, Patrick G Johnston Centre for Cancer Research, 97 Lisburn Rd, Belfast, BT9 7AE Belfast, United Kingdom; 6 Integrated Pathology Unit, Institute of Cancer Research, London, United Kingdom; 7 Department of Cellular Pathology, Belfast Health and Social Care Trust, Royal Victoria Hospital, Grosvenor Road, Belfast, BT12 6BA United Kingdom; 8 School of Medicine, University of St Andrews, North Haugh, St Andrews, KY16 9TF United Kingdom; 9 Wales Cancer Biobank, Cardiff University, 2nd Floor, Room 2LB4 64, A Block, UHW Main Building, Heath Park, Cardiff, CF14 4XN United Kingdom; 10 University of North Carolina at Chapel Hill, Chapel Hill, NC USA; 11 Else Kroener Fresenius Center for Digital Health, Faculty of Medicine and University Hospital Carl Gustav Carus, TUD Dresden University of Technology, 01307 Dresden, Germany; 12 Medical Oncology, National Center for Tumor Diseases (NCT), University Hospital Heidelberg, Heidelberg, Germany

**Keywords:** Biomarkers, Cancer, Computational biology and bioinformatics, Gastroenterology, Oncology

## Abstract

Mismatch repair (MMR) deficiency occurs in 10–20% of colorectal cancer (CRC) cases, leading to microsatellite instability (MSI). Although MSI/MMR testing is critical for CRC management, high costs and long turnaround times limit testing rates and clinical utility, highlighting the need for more accessible, cost-effective alternatives. PANProfiler Colorectal (PPC) is an artificial intelligence (AI)-based biomarker test that determines MSI/MMR status directly from haematoxylin and eosin (H&E)-stained slides. We conducted a blinded, multi-centred validation to assess PPC’s performance against standard testing. The study included 3,576 whole slide images from 1,243 CRC patients across three United Kingdom institutions. PPC produced definitive results for 86.55% of slides, achieving an overall agreement of 93.83%, positive agreement of 92.54%, and negative agreement of 94.02%. PPC accurately determined MSI/MMR status from routine H&E slides, offering a rapid, scalable alternative to conventional diagnostic methods.

## Introduction

The DNA mismatch repair (MMR) system is essential for maintaining DNA integrity by rectifying errors that occur during replication, such as base-base mismatches and insertion-deletion loops, to maintain genomic stability. Four key proteins, MLH1, MSH2, MSH6, and PMS2, are included in this process. If the expression of any of the corresponding genes is impaired, the MMR mechanism can become dysfunctional^1^. When MMR is disrupted, it leads to microsatellite instability (MSI), which is characterised by changes in these repetitive DNA sequences. As such, MSI is a phenotypic indicator of abnormal MMR function^1^. Deficient MMR (dMMR) is evident in 10-20% of colorectal cancers (CRCs)^2–4^. MSI/MMR status can inform the clinical management of CRC patients, with major diagnostic, prognostic, and therapeutic implications, as well as highlighting patients for Lynch syndrome testing^5^. Importantly, MSI-high (MSI-H) and dMMR serve as predictive biomarkers of response to immune checkpoint inhibitors. The pioneering work of Allison and Honjo demonstrated that immunotherapy is particularly effective in CRC with MSI-H or dMMR, with response rates of approximately 50% in metastatic CRC and up to 100% in early-stage cases^6,7^. Therefore, accurate and timely MSI/MMR testing is critical to guide treatment decisions and improve patient outcomes.

Testing for MSI or dMMR is recommended for all CRC patients by the National Institute for Health and Care Excellence (NICE)^8^, the European Society for Medical Oncology (ESMO)^9^, and jointly by the American Society of Clinical Oncology (ASCO) and the College of American Pathologists (CAP)^10^. However, universal MSI/MMR testing is not yet standard practice, with testing rates varying significantly across countries, healthcare settings, and patient demographics^11–13^. Current clinical practice relies on two primary methods for MSI/MMR testing: immunohistochemistry (IHC), which detects loss of nuclear expression of the MMR proteins MLH1, MSH2, MSH6, and PMS2, and polymerase chain reaction (PCR)-based MSI testing, which identifies instability in specific microsatellite loci^14^. Both approaches demonstrate good sensitivity (91–93%) and moderate specificity (79–83%)^15^, yet are limited by practical challenges. While IHC is generally highly concordant, its interpretation can be subject to inter-observer variability in challenging cases^16^, whereas PCR can be affected by poor DNA quality, especially from formalin-fixed, paraffin-embedded (FFPE) samples^17,18^. Both require additional tumour tissue beyond that used for preparing the haematoxylin and eosin (H&E)-stained specimen, specialised infrastructure, and may incur high costs. Combined with pathology workforce shortages and variable turnaround times^13,19,20^, these limitations highlight the need for a rapid, reliable, cost-effective, and widely accessible alternative for MSI/dMMR detection in all CRC patients.

In routine H&E-stained CRC tissue, MSI and dMMR tumours are associated with certain morphological patterns^21^, such as the presence of prominent tumour-infiltrating lymphocytes (TILs) or peritumoral “Crohn’s-like” lymphoid reaction, mucinous differentiation and/or poorly-differentiated morphology^21^. This suggests that MSI/MMR status has distinct histomorphological features identifiable from H&E-stained specimens, which could be leveraged in image-driven algorithms using artificial intelligence. This potential has been demonstrated in multiple studies where AI models were successfully used to determine MSI or MMR status from whole slide images (WSIs) of H&E-stained tissue^21–24^.

**Fig. 1:**
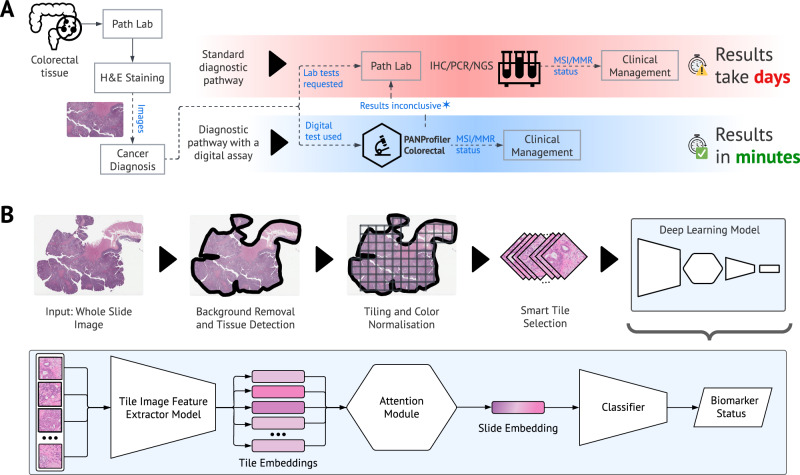
CRC diagnostic pathway with and without PANProfiler Colorectal (PPC). **A** The standard diagnostic pathway for CRC begins with the collection of tissue samples (biopsy or resection). Tissue sections are cut from FFPE blocks, mounted onto glass slides and stained with H&E. These slides are examined by a pathologist, who assesses the presence of tumour and, where present, its grade. Upon confirmation of malignancy, additional testing is performed to assess MSI/MMR status. IHC is commonly used to evaluate the expression of MMR proteins (MLH1, PMS2, MSH2, and MSH6), while PCR/NGS can be used to detect changes in the number of repeats in microsatellite loci, characteristic of MSI. These biomarkers are important for guiding treatment decisions, including the use of immunotherapy. Integrating PPC, a digital test, into routine pathological workflow has the potential to streamline the diagnostic process, reducing the turnaround times from days or weeks to minutes (see also Supplementary Fig. S3), potentially reducing the pressure on histopathology services, and enabling timely treatment decisions. **B** Overview of PPC’s end-to-end deep learning pipeline, including data preprocessing steps, background removal, detection of relevant tissue, tiling and colour normalisation, and selection of tiles for biomarker profiling. PPC’s deep learning architecture consists of a feature extractor and proprietary aggregation, attention and classification modules.

PANProfiler Colorectal (PPC) is an AI-based biomarker test for determining MSI/MMR status from H&E-stained WSIs (Fig. 1). In this paper, we report the performance characteristics of PPC based on a retrospective validation performed in a blinded setting. Data were collected from three UK institutions, ensuring a diverse cohort that enabled assessment of PPC’s robustness and effectiveness across different clinical settings. The results were generated centrally and analysed at St James’s University Hospital (SJUH) in Leeds, UK, without PPC having access to the reference test results at any time of the study. Variability in scanning equipment, image formats, patient demographics, and disease characteristics was considered to reflect real-world conditions. In addition, we discuss PPC’s utility as an AI-based diagnostic test within routine CRC workflows and its potential to streamline the diagnostic processes to enable faster turnaround times.

## Results

### Blinded validation results of PANProfiler colorectal

The key performance characteristics from the blinded validation are given in Table 1. The results demonstrate consistently strong performance of the PPC model across all three independent cohorts. High overall percent agreement (OPA), positive percent agreement (PPA), and negative percent agreement (NPA) were observed, with C-statistics exceeding 0.92 in all cases. The model delivered particularly robust results on the largest cohort (L1-UK-CRC-SVS-1-BLIND). The test replacement rate (TRR) varied across datasets but remained high overall with only 13.45% of tested samples returning an Indeterminate result. These findings support the generalisability and clinical potential of PPC across different institutions and regions in the UK.

**Table 1:** Blinded validation results of PPC by cohort

Cohort	Region	Sample Size (*Unstable* %)	Overall Percent Agreement % (CI)	Positive Percent Agreement % (CI)	Negative Percent Agreement % (CI)	Test Replacement Rate % (CI)	C-statistic
L1-UK- CRC-SVS-1- BLIND	England	3,124 (12.5%)	94.06 (93.11-94.91)	92.45 (89.05-95.05)	94.28 (93.28-95.17)	88.35 (87.17-89.45)	0.9695
W1-UK- CRC-CZI-1- BLIND	Wales	54 (20.4%)	84.31 (71.41-92.98)	100.00 (71.51-100.00)	80.00 (64.35-90.95)	94.44 (84.61-98.84)	0.9205
N1-UK- CRC-SVS-1- BLIND	Northern Ireland	398 (20.6%)	93.31 (89.75-95.92)	91.67 (81.61-97.24)	93.75 (89.74-96.54)	71.36 (66.64-75.75)	0.9670
ALL		3576 (13.5%)	93.83 (92.92-94.65)	92.54 (89.52- 94.91)	94.02 (93.06-94.89)	86.55 (85.39-87.65)	0.9685

C-statistic for all cohorts was computed using a weighted average based on sample size.

### Performance of PANProfiler Colorectal across diverse subpopulations

To demonstrate the generalisability of PPC across different regions within the UK, results from all institutions were analysed together. To this end, we included the I1-UK-CRC-CZI-1-DEV cohort as an additional site to represent Scotland. Since this dataset was validated using 5-fold cross-validation rather than a blind evaluation, it is not included in the main results. The I1-UK-CRC-CZI-1-DEV cohort, consisting of 1,125 WSIs with a prevalence of 13.6%, demonstrated strong performance, achieving an OPA of 86.49% (95% CI: 83.80-88.89%), PPA of 97.41% (95% CI: 92.63-99.46%), NPA of 84.44% (95% CI: 81.34-87.21%), a C-statistic of 0.928, and a TRR of 65.2% (95% CI: 62.29-67.94%). These metrics are within the range of the blind performance results (shown in Table 1), which assessed performance in England, Wales, and Northern Ireland.

### Cohort characteristics

The breakdown of resections and biopsies for development and blind cohorts are provided in Supplementary Table S1. Study population demographics, including age and sex distribution, are detailed in Supplementary Table S2. The mean age across the cohorts ranged from 65.6 to 68.9 years (standard deviation: 11.6 to 12.8 years). Males comprised 52-57% of patients across cohorts, while females accounted for 43-48%, consistent with previously reported figures in the literature^2^. Tumour site distribution is presented in Supplementary Tables S3, with the colon being the most frequently affected site, which aligns with published data^25,26^. Supplementary Table S4 shows the breakdown of histological subtypes across different cohorts. Adenocarcinoma was the predominant histological subtype, observed in 73.24–94.4% of cases, in agreement with prior studies^27^. It was followed by mucinous adenocarcinoma, with a prevalence of 5.56–20.42% across cohorts.

Tumour grade and pathological stage data are summarised in Supplementary Tables S5-S7. These tables also provide the distribution of MSI and dMMR status within each cohort. Moderately differentiated was the most prevalent histological grade, consistent with the literature^27^. Poorly differentiated carcinoma was more frequently observed in patients with MSI-H/dMMR compared to those with non-MSI-H or proficient mismatch repair (pMMR), a finding also supported by earlier studies^21^. Tumour stage varied across cohorts, with stage II and III being the most common. MSI-H/dMMR prevalence was highest in stage II cases. Compared to the published literature, this study observed a lower proportion of stage IV diagnoses and a higher proportion of stage II cases^26,28^. However, the association between lower tumour stage and increased MSI-H/dMMR prevalence is consistent with previous findings^29^.

### Impact of backbone model on performance

The self-supervised learning (SSL)-pretrained backbone outperformed the ImageNet backbone across all metrics in the five-fold cross-validation on the L1-UK-CRC-SVS-1-DEV cohort (see Supplementary Table S8 and Supplementary Fig. S1). It achieved a higher C-statistic (0.96 vs. 0.93), OPA (95.25% vs. 91.48%), PPA (95.51% vs. 87.78%), and NPA (95.19% vs. 92.39%). Additionally, the TRR was slightly higher for SSL (84.03% vs. 83.12%). Consequently, the SSL-based model, demonstrating optimal performance, was selected for the blinded validation study.

### Explainability and interpretability of deep learning models

**Fig. 2:**
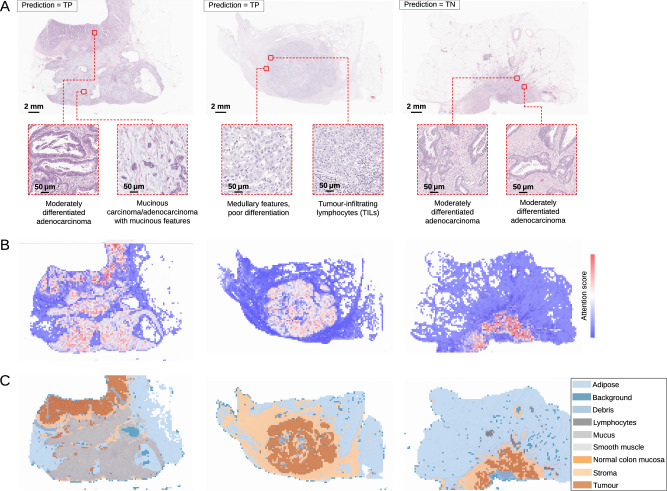
Explainability and interpretability visualisations for images correctly classified by the model, where two true positive (TP) and one true negative (TN) cases from the L1-UK-CRC-SVS-1-BLIND cohort are shown. **A** Original WSI and two regions of interest (ROIs) beneath it (selected from different regions indicated by the red boxes). **B** Attention scores with darker hues of red indicating high attention and blue indicating low attention. **C** Annotated regions based on a classification of the tissue, including adipose, background, debris, lymphocytes, mucus, smooth muscle, normal colon mucosa, stroma, and tumour.

**Fig. 3:**
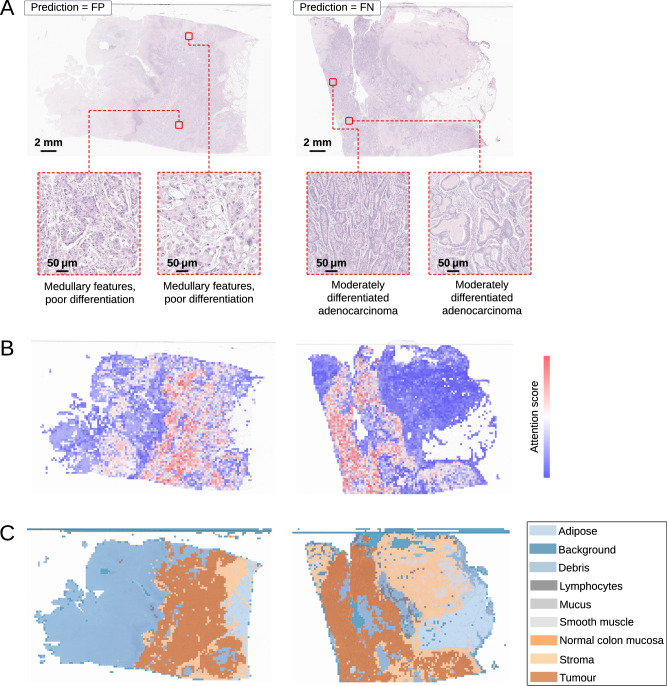
Explainability and interpretability visualisations for images incorrectly classified by the model, where one false positive (FP) and one false negative (FN) images from the L1-UK-CRC -SVS-1-BLIND cohort are shown. **A** Original WSI and two ROIs beneath it (selected from different regions indicated by the red boxes). **B** Attention scores with darker hues of red indicating high attention and blue indicating low attention. **C** Annotated regions based on a classification of the tissue, including adipose, background, debris, lymphocytes, mucus, smooth muscle, normal colon mucosa, stroma, and tumour.

We unblinded and analysed 61 images from the L1-UK-CRC-SVS-1-BLIND cohort, with a breakdown of 16 true positives (TP), 18 true negatives (TN), 7 false positives (FP), 6 false negatives (FN), 8 indeterminate negatives (IN), and 6 indeterminate positives (IP). In Figs. 2, 3, and Supplementary Fig. S2, example images demonstrate how our models direct their attention, by overlaying the “attention heatmaps” (**B**) onto the original WSIs **(A)**. The same panel also highlights the regions of interest selected based on the model’s attention.

To analyse the predictions correctly classified by the model, we visualised the heatmaps for TP and TN cases. For TP cases, regions with high attention scores predominantly aligned with tumour tissue, such as poorly/moderately differentiated areas with medullary features, TILs, and adenocarcinoma with mucinous features (Fig. 2, A-B). In TN cases (Fig. 2, right), the model’s attention was directed toward tumour regions featuring moderately differentiated adenocarcinomas.

To analyse the predictions incorrectly classified by the model, we visualised the heatmaps for FP and FN cases (Fig. 3). FP predictions were focused on poorly differentiated regions, which could explain the misclassification, as poor differentiation is often linked to MSI-H/dMMR tumours (Fig. 3, left). For a FN case (Fig. 3, right), the model concentrated on regions of moderately differentiated adenocarcinoma, likely contributing to the misclassification.

To investigate the model’s indeterminate predictions, we visualised heatmaps for the IN and IP cases (Supplementary Fig. S2). Our analysis suggests that indeterminate predictions may arise from conflicting or ambiguous morphological signals. For instance, some non-MSI-H/pMMR (IN) cases presented with features typically associated with MSI-H/dMMR status (e.g., poor differentiation, TILs, mucinous features), while others were confounded by factors like limited tumour tissue or contained non-specific features such as moderate differentiation. Similarly, most indeterminate MSI-H/dMMR (IP) cases exhibited a substantial degree of well or moderate differentiation.

Overall, increased attention is observed on biologically meaningful morphological features, such as poor differentiation, TILs, and mucinous characteristics, which are well-documented as being associated with MSI-H/dMMR. By contrast, well-differentiated tumours are commonly linked to non-MSI-H/pMMR. Moderate differentiation, on the other hand, is not a reliable discriminator of MSI/MMR status, as it is observed in both MSI-H/dMMR and non-MSI-H/pMMR tumours^23,24,30–33^.

**Fig. 4:**
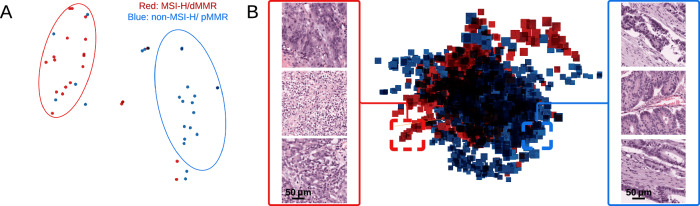
Visualisation of the embedding space at slide and patch levels. The embedding space of 61 whole slide images (WSIs) from the L1-UK-CRC-SVS-1-BLIND cohort is visualised at both slide (**A**) and patch (**B**) levels. MSI-H/dMMR and non-MSI-H/pMMR images and patches are shown in red and blue, respectively. The slide level embeddings (**A**) are visualised using 2D t-SNE, while the top 100 scoring attention tiles per WSI (**B**) are visualised using 3D t-SNE. An example of three closest MSI-H/dMMR and non-MSI-H/pMMR patches (based on cosine distance) is shown, taken from regions indicated by red and blue boxes, respectively.

The model’s embedding space was also evaluated to determine whether MSI-H/dMMR and non-MSI-H/pMMR cases could be separated. Embedding spaces were visualised at both the slide level (Fig. 4A) and patch level (Fig. 4B). The slide-level (classification) embedding space (Fig. 4A) demonstrated clear separation between the MSI-H/dMMR and non-MSI-H/pMMR classes. At the patch level, distinct regions for each class were observed, with patches exhibiting similar morphology positioned closer together in the embedding space. For example, MSI-H/dMMR patches in one region formed clusters characterised by poorly differentiated adenocarcinoma, while another non-MSI-H/pMMR cluster of patches were associated with moderately differentiated adenocarcinoma.

## Discussion

This study evaluated the performance of AI-based PPC compared to standard pathology MSI/MMR testing in a retrospective, blinded setting. High PPA and NPA demonstrated that PPC accurately identified both MSI-high/dMMR (*Unstable*) and non-MSI-high/pMMR (*Stable*) samples. Additionally, of 3576 WSIs, PPC returned definite results (TRR) for 86.55% of images, indicating a potential significant reduction in the number of standard pathology tests required.

Many other studies have evaluated AI-driven methods for determining MSI/MMR status from WSIs^34–39^. These demonstrate the feasibility of AI-derived image features for MSI/MMR detection. However, most have focused on screening, where the primary target was identifying cases not requiring further molecular or IHC testing. For instance, one study^33^ conducted a blinded validation on 600 WSIs, reporting an area under the receiver operating characteristic curve (AUC, i.e. the C-statistic) of 88% and a sensitivity (i.e. PPA) of 96-98% with a TRR of 46-47%. Other studies reported comparable or worse performance. For example, one study^40^ achieved TRRs of 44.12% for resections and 52.73% for biopsies with a sensitivity of 95%. Moreover, another approach for MSI screening^41^ introduced a deep learning (DL)-based classifier capable of replacing testing for only 40% of CRC cases.

Other AI-based approaches have addressed the problem from a diagnostic perspective, aiming to identify both MSI-high/dMMR and non-MSI-high/pMMR cases. One diagnostic approach^42^ validated on 355 resections and 341 biopsies, reported AUCs of 84.57% and 76.79%, sensitivity scores of 90.91% and 92.31%, specificity (i.e. NPA) scores of 95.13% and 95.36%, and TRRs of 56.06% and 51.03%, respectively. Finally, a recent large-scale study^23^ by Wagner et al. reported sensitivity scores of 98-99% and specificity scores of 44-56%. While there exist key differences in model architecture and training data characteristics, our study’s higher specificity fundamentally stems from different clinical goals and model designs. The Wagner et al. model was developed for pre-screening, where a trade-off with specificity is accepted to maximise sensitivity. In contrast, our tool is designed for diagnostic use, requiring both high sensitivity and specificity. We achieve this by implementing a three-tiered classification system (‘Stable’, ‘Unstable’, ‘Indeterminate’), which allows the model to make definitive calls with higher confidence by classifying ambiguous cases as ‘Indeterminate’. This methodological choice is the primary driver of our higher specificity. Consequently, in contrast to previous methods, PPC demonstrates remarkable diagnostic utility by maintaining both high sensitivity and specificity. This also translates to great practical application, with an overall TRR of 86.55%.

We evaluated the safety of PPC by comparing its performance to standard MSI/MMR detection tests, including PCR for MSI testing and IHC for MMR deficiency, based on results from clinical studies in the literature. This is critical to show that PPC yields similar risk in the context of false negative and false positive results, as such there would be no greater risk of adverse effects compared to the current standard of care. Aggregated data from multiple studies primarily across the US and Europe^15^ indicate that MSI testing (n = 3,476) has a sensitivity (PPA) of 93% (95% CI: 87-96%) and a specificity (NPA) of 79% (95% CI: 70-86%), while MMR testing (n = 3,091) shows a sensitivity of 91% (95% CI: 85-95%), and a specificity of 83% (95% CI: 77-88%). The National Institute for Health and Care Excellence (NICE), England, reported similar figures^8^ for MSI testing (sensitivity: 91.3% [95% CI: 42.6-99.3%], specificity: 83.7% [95% CI: 63.8-93.7%]) and IHC-based MMR testing (sensitivity: 96.2% [95% CI: 69.4-99.6%], specificity: 88.4% [95% CI: 79.0-94.0%]). Recent studies report that concordance between MSI testing and MMR IHC is increasing, especially when sufficient tumour DNA is present and unusual IHC patterns are taken into account during MMR testing^43^. PPC’s performance appears comparable to standard pathology tests, however, direct head-to-head comparisons with other studies are often challenging due to variations in patient cohorts, experimental design, preanalytical protocols and the specific definitions of outcomes. Therefore, any comparison of our results to other published data should be interpreted with caution.

The integration of PPC into pathological workflows could offer significant advantages, particularly in its ability to streamline diagnostic processes by minimising reliance on time-intensive assays currently used as the standard for biomarker testing. This reduction in testing could assist users, such as pathologists and laboratory professionals, in managing growing workloads and addressing staffing shortages^19,20^. By contrast, PPC can generate biomarker results within minutes (Supplementary Fig. S3), enabling pathology reports to be finalised simultaneously during the histological assessment. Other potential benefits of PPC include lowering the diagnostic burden on pathologists and lab professionals, allowing them to dedicate more attention to complex cases. A digital test based on existing H&E-stained slides offers positive environmental benefits by reducing the need for additional wet laboratory procedures, minimising the use of toxic chemicals and consumables common in conventional diagnostics. By streamlining workflows and reducing the reliance on standard laboratory testing, PPC also has the potential to deliver timely results and greater cost efficiency for healthcare providers. Future work will include a health economics analysis to evaluate its cost-effectiveness in real-world clinical environments. In parallel, usability studies with target users will assess the impact of PPC on user experience and workflow integration.

While PPC’s overall performance demonstrated significant promise, certain aspects warrant additional exploration to improve the AI-based test. Approximately 14% of samples returned an Indeterminate result, which may be partially due to preanalytical factors such as suboptimal tissue processing, staining or slide digitisation. Standardising and optimising these workflows will be key to increasing the proportion of assessable cases and improving clinical usability. Indeterminate outcomes may also reflect model uncertainty driven by morphological inconsistencies. Our visual analysis of indeterminate cases showed they often contained conflicting features, such as non-MSI cases presenting with MSI-like features (e.g., TILs and poor differentiation), non-specific features like moderate differentiation, or confounding factors like limited tumour tissue, leading to an indeterminate result (Supplementary Fig. S2). Expanding the training dataset to include more diverse, representative cases could improve the model’s ability to resolve such differences. In addition, a deeper investigation into the variability of validation metrics such as TRR is needed to better understand and refine PPC’s performance across different cohorts. All institutions in the study were based in the UK, providing a strong foundation within a well-characterised healthcare system. To build on these findings and support broader applicability, future validation will benefit from including samples from other regions and more diverse demographics. While this retrospective study offers valuable insights, prospective studies in clinical environments will be key to further demonstrating PPC’s performance and utility in real-world clinical settings. Such studies would provide a critical understanding of PPC’s integration into routine pathology workflows and assess its impact on patient outcomes. Finally, this study utilised mostly resected tissue samples (Supplementary Table S1). Since biopsies are more commonly used in routine clinical practice, future validation efforts will expand to include biopsy samples, further supporting the test’s real-world applicability.

Taken together, these findings point to the potential of PPC as a proficient diagnostic tool capable of comparable levels of performance to current standard-of-care testing. This paves the way towards improved diagnostics, ensuring more accurate results and leading to the timely establishment of MSI/MMR status for CRC cases.

## Methods

### Cohort characteristics for blinded validation

A total of 3576 WSIs from three different UK cohorts were used for the multi-centred, blinded validation of PPC (Table 2). Only WSIs derived from FFPE blocks of primary CRC were included in the analysis. WSIs of fresh frozen samples, metastatic tumours from non-colorectal sites, and those with less than 10% tumour content were excluded. Additionally, WSIs failing visual assessment and quality control by pathologists due to excessive tissue folds, air bubbles, pen marks, adhesive tape, out-of-focus regions, pixel artefacts or other digital distortions were also excluded. The number of excluded images (n = 1234) accounted for 25.65% of the images originally acquired from the three institutions (n = 4,810). The WSIs were acquired using different scanners and stored in multiple image formats. MSI-H/dMMR prevalence ranged from 12.52 to 20.60%. This is comparable to global estimates^2–4^, which serve as the primary reference for clinical practice in the UK. The make-up of each independent cohort was as follows:

**Table 2:** Details of the cohorts used in the multi-centred, blinded validation study for PANProfiler Colorectal

Cohort	Images received	Images with verified MSI/MMR status^a^	Number of patients with verified MSI/MMR status^a^	MSI-H/dMMR prevalence %
L1-UK-CRC-SVS-1- BLIND	3,884	3,124	791	12.52
W1-UK-CRC-CZI-1- BLIND	510	54	54	20.37
N1-UK-CRC-SVS-1- BLIND	416	398	398	20.60
**Combined Total**	**4810**	**3576**	**1243**	**13.53**

^a^WSIs were excluded based on the study’s inclusion and exclusion criteria (see **Methods: Cohort Characteristics)**, including pathological assessment and the availability of corresponding MSI/MMR status.

Bold values are the combined totals across all cohorts.

#### L1-UK-CRC-SVS-1-BLIND

This cohort comprised 3,884 archival CRC images, sourced from St James’s University Hospital (SJUH) in Leeds, UK, of which 3,124 had MSI/MMR status (391 *Unstable* and 2,733 *Stable*). Tissue samples were scanned at 20/40x magnification using Leica Aperio GT 450 DX, AT2 or Scanscope CS scanners and stored in SVS format.

#### W1-UK-CRC-CZI-1-BLIND

This cohort was composed of a total of 510 WSIs, sourced from Wales Cancer Biobank (WCB) in Cardiff, UK, of which 54 had MSI/MMR status (11 *Unstable* and 43 *Stable*). Tissue samples were scanned at 40x magnification and stored in CZI format.

#### N1-UK-CRC-SVS-1-BLIND

This cohort comprised 398 WSIs collected from Northern Ireland Biobank (NIB) in Belfast, UK (82 *Unstable*, 316 *Stable*). Tissue samples were scanned at 20/40x magnification using a Leica Aperio AT2 scanner and stored in SVS format.

### Cohort characteristics for development and calibration

A total of 2502 WSIs of FFPE CRC specimens from four cohorts were used for development, internal validation, and calibration of PPC (Table 3). While samples from the blinded validation and development/calibration cohorts may originate from the same institution, there is no case overlap between these distinct cohorts. Each case included in the blinded validation cohort is unique and has not been used in the development or calibration phases of the model. The make-up of each cohort was as follows:

**Table 3:** Details of the cohorts used for development, internal validation and calibration of PANProfiler Colorectal

Cohort	Images received	Images with verified MSI/MMR status^a^	Number of patients with verified MSI/MMR status^a^	MSI-H/dMMR prevalence %
T1-US-CRC-SVS-1-DEV	625	563	554	13.85
I1-UK-CRC-CZI-1-DEV	1196	1156	1011	13.75
L1-UK-CRC-SVS-1-DEV	551	551	142	19.78
N1-UK-CRC-SVS-1-DEV	130	130	130	20.77
**Combined Total**	**2502**	**2400**	**1837**	**15.54**

^a^WSIs were excluded based on the study’s inclusion and exclusion criteria (see **Methods: Cohort Characteristics)**, including pathological assessment and the availability of corresponding MSI/MMR status.

Bold values are the combined totals across all cohorts.

#### T1-US-CRC-SVS-1-DEV

This cohort consisted of 563 WSIs (78 *Unstable* and 485 *Stable*), acquired from The Cancer Genome Atlas (TCGA) Colon (COAD) and rectal (READ) adenocarcinoma studies (https://portal.gdc.cancer.gov/). It was used for model development and internal validation. Tissue samples were scanned at 20/40x magnification and stored in SVS format.

#### I1-UK-CRC-CZI-1-DEV

This cohort consisted of 1,156 WSIs (159 *Unstable* and 997 *Stable*), sourced from the Industrial Centre for Artificial Intelligence Research in Digital Diagnostics (iCAIRD) in Scotland, UK. It was used for model development and internal validation. Tissue samples were scanned using Zeiss Axioscan at 40x magnification and stored in CZI format.

#### L1-UK-CRC-SVS-1-DEV

This cohort consisted of 551 WSIs (109 *Unstable* and 442 *Stable*) collected from SJUH in Leeds. It was used for internal validation and calibration. Tissue samples were scanned at 20/40x magnification using Leica Aperio GT 450 DX, AT2 or Scanscope CS scanners and stored in SVS format.

#### N1-UK-CRC-SVS-1-DEV

This cohort consisted of 130 WSIs (27 *Unstable* and 103 *Stable*) collected from NIB. It was used for internal validation and calibration. Tissue samples were scanned at 20/40x magnification using a Leica Aperio AT2 scanner and stored in SVS format.

### Overview of PANProfiler colorectal

PPC is an AI-driven biomarker test developed to determine MSI/MMR status from digitally scanned H&E-stained slides of FFPE primary CRC specimens. PPC classifies MSI/MMR status as *‘Unstable*’, *Stable*’ or *‘Indeterminate’*. *‘Unstable’* was defined as MSI-high/dMMR (i.e. positive class), while *‘Stable’* corresponded to pMMR/non-MSI-high (i.e. negative class). An *‘Indeterminate’* result was returned when the test could not confidently determine MSI/MMR status.

### Performance characteristics and statistical procedures

PPC’s performance was evaluated through a comprehensive agreement analysis with standard pathology tests, employing four key metrics: overall percent agreement (OPA), positive percent agreement (PPA), negative percent agreement (NPA) and test replacement rate (TRR), which measured the percentage of cases for which PPC was able to provide a definitive result. In addition, the C-statistic, equivalent to the area under the receiver operating characteristic curve (AUC), was reported^44,45^. Formal definitions of all performance metrics are provided in Supplementary Tables S9 and S10.

Based on the expected prevalence of MSI-H/dMMR in colorectal cancer (10-20%), a power analysis determined that a minimum of 140-245 cases would be required to achieve a 95% confidence level with a 5% margin of error^46^. The sample size used in this study therefore exceeds the threshold necessary to ensure adequate statistical power.

### Artificial intelligence for establishing MSI/MMR status

PPC is powered by AI and digital pathology. The platform relies on a series of DL methods, a subset of AI that enables computers to learn patterns from large datasets. Specifically, it can identify complex histomorphological features in relevant parts of H&E-stained WSIs of CRC and use them to determine MSI/MMR status^47^. Figure 1 illustrates the core preprocessing and DL components that underlie PPC and how they are connected in an end-to-end pipeline.

A DL model was trained to predict MSI/MMR status as follows. In the first step, input WSIs were subdivided into image patches (i.e. tiles) of 256×256 pixels at a resolution of 1.0 microns per pixel (MPP). Proprietary filtering algorithms were used to eliminate the background tiles and detect tissue. A WSI was discarded from analysis if it contained fewer than 10 tiles after the filtering process. Colour normalisation^48^ was applied to the remaining tiles before they were assigned with a reference biomarker status.

The selected pre-processed tiles were then used in an end-to-end DL pipeline. This pipeline consisted of the following components: (1) a smart sampling module used for extracting groups of tiles that are spatially adjacent (referred to as *windows*); (2) a feature extractor CNN (i.e., an encoder) for learning unique feature representations for each tile; (3) an attention module that aggregated the tile embedding into a slide level embedding, weighting the features based on their relevance (using the attention scores) for the target biomarker. The attention module was finally connected to a classification layer that made the final diagnostic call on the biomarker status.

### Explainability and interpretability of deep learning models

Understanding the internal mechanisms of DL models can provide insights into how they detect visual patterns in tissue WSIs that may be correlated with biological signals. Visualising the “black-box” nature of these models offers valuable insights into the explainability of their outputs. A common approach to visualising the spatial regions critical for determining biomarker status is overlaying tile-level attention on WSIs to create spatial heatmaps. These heatmaps highlight the areas of the input image that have the strongest influence on the biomarker result. DL models can identify morphological patterns or visual features that are not immediately visible to the human eyes and correlate them with the existence (or absence) of biological signals, such as biomarker expression. We visualise the heatmaps by overlaying the model attention onto the WSI in Figs. 2 and 3.

In addition, to enhance the interpretability of the models, a DL model was developed and trained to classify various tissue types in CRC samples using the NCT-CRC-HE-100K dataset^49^. The model categorised tissues into the following classes: adipose, background, debris, lymphocytes, mucus, smooth muscle, normal colon mucosa, stroma, and tumour. This model was used to confirm whether the attention of the primary models was directed toward relevant morphological regions by overlaying the classification maps onto the WSIs and comparing them with the attention heatmaps.

To gain deeper insights into the PPC model, its embedding space, a lower-dimensional vector representation of image patches or whole slides, was visualised. Patch-level embeddings were obtained by processing each WSI through the model’s feature extractor, and the resulting vectors for individual patches were stored. These patch embeddings were then aggregated into slide-level embeddings using the model’s attention layer and saved for each WSI. The embeddings were visualised in TensorBoard using t-distributed Stochastic Neighbour Embedding (t-SNE), a technique for visualising high-dimensional data in two or three dimensions, with data points organised by their respective classes (Fig. 4). To measure similarity between embeddings, the cosine distance, a metric based on the angle between two vectors, was used. For this analysis, a subset of 61 WSIs from the L1-UK-CRC-SVS-1-BLIND cohort was unblinded, comprising 28 samples with MSI-H/dMMR and 33 with non-MSI-H/pMMR.

### Development and validation strategies

We utilised an SSL framework (Mocov2) for pre-training base models, allowing them to learn feature representations from unlabelled data and develop robustness for diverse tasks^50^. Using the pre-trained SSL model as a backbone, a model was further fine-tuned through supervised learning with labels derived from reference MSI/MMR results. The SSL training datasets consisted of 588 WSIs from the T1-US-CRC-SVS-1-DEV cohort. The development datasets (Table 3) consisted of 1,719 WSIs (237 *Unstable*, 1,482 *Stable* WSIs), including T1-US-CRC-SVS-1-DEV (n = 563, with 78 *Unstable* and 485 *Stable* WSIs), and I1-UK-CRC-CZI-1-DEV (n = 1,156, with 159 *Unstable*, and 997 *Stable* WSIs).

To determine the best model to be deployed for blinded validation, different models were evaluated on the L1-UK-CRC-SVS-1-DEV cohort using cross-validation. To this end, this cohort was divided into five equal-sized folds. A model was fine-tuned, validated, and tested five times, cycling through a few fold combinations. This ensured that the models were thoroughly evaluated over the entirety of a cohort while minimising the risk of overfitting and providing a robust assessment of model performance.

Calibration refers to finding the most optimal decision thresholds for a model. These thresholds help the model operate safely by allowing an ‘Indeterminate’ output when no definitive result can be provided. PPC’s safety and effectiveness were maintained across different sites by calibrating cohort-specific thresholds for each biomarker. The decision boundaries identified during the calibration process were applied to compute the final model outputs, which were then used to calculate performance metrics on the test set for each cohort (see **Performance characteristics** for details).

After determining the optimal configuration, PPC underwent separate calibrations on two cohorts before the blinded validation phase. Specifically, calibration was performed on the L1-UK-CRC-SVS-1-DEV cohort, consisting of 551 WSIs (109 *Unstable* and 442 *Stable* images), and the N1-UK-CRC-SVS-1-DEV cohort, which included 130 WSIs (27 *Unstable* and 103 *Stable*). No calibration was performed for the WCB cohort (W1-UK-CRC-CZI-1-BLIND), due to a low sample size.

### Blinded validation

Authors from the University of Leeds conducted the blinded validation across all three cohorts. MSI/MMR status associated with blinded cohorts was not disclosed at any stage to the analysts. WSIs were analysed using PPC, generating test results classified as Stable, Unstable, or Indeterminate. These results were then shared with the clinical research team performing the blinded validation, who constructed confusion matrices and calculated performance metrics.

## Supplementary information

Supplementary Materials

## Data Availability

The Cancer Genome Atlas (TCGA) used for training is publicly available from the following link: [https://portal.gdc.cancer.gov/]. The additional datasets used for training and blinded validation were obtained through agreements between Panakeia Technologies Limited and the respective data providers and are not publicly available.

## Code availability

The software and AI algorithms used in the current study are Panakeia Technologies Limited proprietary IP and cannot be shared.

## References

[1] Pećina-Šlaus, N., Kafka, A., Salamon, I. & Bukovac, A. Mismatch Repair Pathway, Genome Stability and Cancer. *Front. Mol. Biosci*. **7**, (2020). 10.3389/fmolb.2020.00122 PMC7332687 32671096

[2] Koopman, M. et al. Deficient mismatch repair system in patients with sporadic advanced colorectal cancer. *Br. J. Cancer* **100**, 266–273 (2009). 19165197 10.1038/sj.bjc.6604867 PMC2634718

[3] Kang, Y.-J. et al. A scoping review and meta-analysis on the prevalence of pan-tumour biomarkers (dMMR, MSI, high TMB) in different solid tumours. *Sci. Rep.* **12**, 20495 (2022). 36443366 10.1038/s41598-022-23319-1 PMC9705554

[4] Lorenzi, M., Amonkar, M., Zhang, J., Mehta, S. & Liaw, K.-L. Epidemiology of Microsatellite Instability High (MSI-H) and Deficient Mismatch Repair (dMMR) in Solid Tumors: A Structured Literature Review. *J. Oncol.* **2020**, 1807929 (2020).

[5] Ma, J., Setton, J., Lee, N. Y., Riaz, N. & Powell, S. N. The therapeutic significance of mutational signatures from DNA repair deficiency in cancer. *Nat. Commun.* **9**, 3292 (2018). 30120226 10.1038/s41467-018-05228-y PMC6098043

[6] Cercek, A. et al. PD-1 blockade in mismatch repair–deficient, locally advanced rectal cancer. *N. Engl. J. Med.* **386**, 2363–2376 (2022). 35660797 10.1056/NEJMoa2201445 PMC9492301

[7] Morse, M. A., Hochster, H. & Benson, A. Perspectives on treatment of metastatic colorectal cancer with immune checkpoint inhibitor therapy. *Oncologist* **25**, 33–45 (2020). 31383813 10.1634/theoncologist.2019-0176 PMC6964145

[8] Molecular testing strategies for Lynch syndrome in people with colorectal cancer. *Nice* https://www.nice.org.uk/guidance/dg27.

[9] Stjepanovic, N. et al. Hereditary gastrointestinal cancers: ESMO Clinical Practice Guidelines for diagnosis, treatment and follow-up. *Ann. Oncol.* **30**, 1558–1571 (2019). 31378807 10.1093/annonc/mdz233

[10] Vikas, P. et al. Mismatch repair and microsatellite instability testing for immune checkpoint inhibitor therapy: ASCO Endorsement of College of American Pathologists Guideline. *J. Clin. Oncol.* **41**, 1943–1948 (2023). 36603179 10.1200/JCO.22.02462

[11] Garcia-Carbonero, R. et al. Real-world study on microsatellite instability and mismatch repair deficiency testing patterns among patients with metastatic colorectal cancer in Spain. *Clin. Transl. Oncol.* **26**, 864–871 (2024). 37651021 10.1007/s12094-023-03309-z PMC10981578

[12] Papke, D. J., Lindeman, N. I., Schrag, D. & Iorgulescu, J. B. Underutilization of guideline-recommended mismatch repair/microsatellite instability biomarker testing in advanced colorectal cancer. *Cancer Epidemiol. Biomark. Prev. Publ. Am. Assoc. Cancer Res. Cosponsored Am. Soc. Prev. Oncol.* **31**, 1746–1751 (2022). 10.1158/1055-9965.EPI-22-0279 PMC9444979 35767976

[13] Echle, A. et al. Clinical-grade detection of microsatellite instability in colorectal tumors by deep learning. *Gastroenterology* **159**, 1406–1416.e11 (2020). 32562722 10.1053/j.gastro.2020.06.021 PMC7578071

[14] Chen, J. et al. Microsatellite status detection of colorectal cancer: evaluation of inconsistency between PCR and IHC. *J. Cancer* **14**, 1132–1140 (2023). 37215453 10.7150/jca.81675 PMC10197936

[15] Ladabaum, U., Ford, J. M., Martel, M. & Barkun, A. N. American Gastroenterological Association technical review on the diagnosis and management of lynch syndrome. *Gastroenterology* **149**, 783–813.e20 (2015). 26226576 10.1053/j.gastro.2015.07.037

[16] Yu, F., Makrigiorgos, A., Leong, K. W. & Makrigiorgos, G. M. Sensitive detection of microsatellite instability in tissues and liquid biopsies: Recent developments and updates. *Comput. Struct. Biotechnol. J.* **19**, 4931–4940 (2021). 34527197 10.1016/j.csbj.2021.08.037 PMC8433064

[17] Sieben, N. L. G., Ter Haar, N. T., Cornelisse, C. J., Jan Fleuren, G. & Cleton-Jansensen, A.-M. PCR artifacts in LOH and MSI analysis of microdissected tumor cells. *Hum. Pathol.* **31**, 1414–1419 (2000). 11112218

[18] Monument, M. J., Lessnick, S. L., Schiffman, J. D. & Randall, R. L. Microsatellite Instability in Sarcoma: Fact or Fiction?. *Int. Sch. Res. Not.* **2012**, 473146 (2012). 10.5402/2012/473146 PMC3564276 23401795

[19] Walsh, E. & Orsi, N. M. The current troubled state of the global pathology workforce: a concise review. *Diagn. Pathol.* **19**, 163 (2024). 39709433 10.1186/s13000-024-01590-2 PMC11662708

[20] Robboy, S. J. et al. The Pathologist Workforce in the United States: II. An Interactive Modeling Tool for Analyzing Future Qualitative and Quantitative Staffing Demands for Services. *Arch. Pathol. Lab. Med.* **139**, 1413–1430 (2015). 26516939 10.5858/arpa.2014-0559-OA

[21] Greenson, J. K. et al. Pathologic predictors of microsatellite instability in colorectal cancer. *Am. J. Surg. Pathol.* **33**, 126–133 (2009). 18830122 10.1097/PAS.0b013e31817ec2b1 PMC3500028

[22] Chang, X. et al. Predicting colorectal cancer microsatellite instability with a self-attention-enabled convolutional neural network. *Cell Rep. Med.* **4**, 100914 (2023). 36720223 10.1016/j.xcrm.2022.100914 PMC9975100

[23] Wagner, S. J. et al. Transformer-based biomarker prediction from colorectal cancer histology: A large-scale multicentric study. *Cancer Cell* **41**, 1650–1661.e4 (2023). 37652006 10.1016/j.ccell.2023.08.002 PMC10507381

[24] Kather, J. N. et al. Deep learning can predict microsatellite instability directly from histology in gastrointestinal cancer. *Nat. Med.* **25**, 1054–1056 (2019). 31160815 10.1038/s41591-019-0462-y PMC7423299

[25] White, A. et al. A review of sex-related differences in colorectal cancer incidence, screening uptake, routes to diagnosis, cancer stage and survival in the UK. *BMC Cancer* **18**, 906 (2018). 30236083 10.1186/s12885-018-4786-7 PMC6149054

[26] Himbert, C. et al. Clinical characteristics and outcomes of colorectal cancer in the colocare study: differences by age of onset. *Cancers* **13**, 3817 (2021). 34359718 10.3390/cancers13153817 PMC8345133

[27] Fleming, M., Ravula, S., Tatishchev, S. F. & Wang, H. L. Colorectal carcinoma: Pathologic aspects. *J. Gastrointest. Oncol.* **3**, 153–173 (2012). 22943008 10.3978/j.issn.2078-6891.2012.030 PMC3418538

[28] McPhail, S., Johnson, S., Greenberg, D., Peake, M. & Rous, B. Stage at diagnosis and early mortality from cancer in England. *Br. J. Cancer* **112**, S108–S115 (2015). 25734389 10.1038/bjc.2015.49 PMC4385983

[29] Gutierrez, C., Ogino, S., Meyerhardt, J. A. & Iorgulescu, J. B. The Prevalence and Prognosis of Microsatellite Instability-High/Mismatch Repair-Deficient Colorectal Adenocarcinomas in the United States. *JCO Precis. Oncol*. e2200179 10.1200/PO.22.00179. (2023). 10.1200/PO.22.00179 PMC9928756 36716414

[30] Cao, R. et al. Development and interpretation of a pathomics-based model for the prediction of microsatellite instability in Colorectal Cancer. *Theranostics* **10**, 11080–11091 (2020). 33042271 10.7150/thno.49864 PMC7532670

[31] Nguyen, H.-G. et al. Image-based assessment of extracellular mucin-to-tumor area predicts consensus molecular subtypes (CMS) in colorectal cancer. *Mod. Pathol.* **35**, 240–248 (2022). 34475526 10.1038/s41379-021-00894-8 PMC8786661

[32] Adib, E. et al. Deep learning–powered analysis of tumor-infiltrating lymphocytes (TILs) in colorectal cancer. *J. Clin. Oncol.* **43**, 288–288 (2025).

[33] Saillard, C. et al. Validation of MSIntuit as an AI-based pre-screening tool for MSI detection from colorectal cancer histology slides. *Nat. Commun.* **14**, 6695 (2023). 37932267 10.1038/s41467-023-42453-6 PMC10628260

[34] Hezi, H., Gelber, M., Balabanov, A., Maruvka, Y. E. & Freiman, M. CIMIL-CRC: A clinically-informed multiple instance learning framework for patient-level colorectal cancer molecular subtypes classification from H&E stained images. *Comput. Methods Prog. Biomed.* **259**, 108513 (2025). 10.1016/j.cmpb.2024.108513 39581068

[35] Gustav, M. et al. Deep learning for dual detection of microsatellite instability and POLE mutations in colorectal cancer histopathology | npj Precision Oncology. *Npj Precis. Oncol*. **8**, (2024). 10.1038/s41698-024-00592-z PMC11116442 38783059

[36] He, B. et al. Development of a Multimodal Deep Learning Model for Predicting Microsatellite Instability in Colorectal Cancer by Integrating Histopathological Images and Clinical Data. Preprint at 10.21203/rs.3.rs-4200523/v1 (2024).

[37] Raza, M., Awan, R., Bashir, R. M. S., Qaiser, T. & Rajpoot, N. M. Dual attention model with reinforcement learning for classification of histology whole-slide images. *Comput. Med. Imaging Graph.* **118**, 102466 (2024). 39579453 10.1016/j.compmedimag.2024.102466

[38] Nowak, M. et al. Single-cell AI-based detection and prognostic and predictive value of DNA mismatch repair deficiency in colorectal cancer. *Cell Rep. Med.* **5**, 101727 (2024). 39293403 10.1016/j.xcrm.2024.101727 PMC11525017

[39] Lo, C.-M., Jiang, J.-K. & Lin, C.-C. Detecting microsatellite instability in colorectal cancer using Transformer-based colonoscopy image classification and retrieval. *PloS One* **19**, e0292277 (2024). 38271352 10.1371/journal.pone.0292277 PMC10810505

[40] Echle, A. et al. Artificial intelligence for detection of microsatellite instability in colorectal cancer—a multicentric analysis of a pre-screening tool for clinical application. *ESMO Open* **7**, 100400 (2022). 35247870 10.1016/j.esmoop.2022.100400 PMC9058894

[41] Lee, S. H., Song, I. H. & Jang, H.-J. Feasibility of deep learning-based fully automated classification of microsatellite instability in tissue slides of colorectal cancer. *Int. J. Cancer* **149**, 728–740 (2021). 33851412 10.1002/ijc.33599

[42] Clinical actionability of triaging DNA mismatch repair deficient colorectal cancer from biopsy samples using deep learning - eBioMedicine. *The Lancet* **81**, (2022). 10.1016/j.ebiom.2022.104120 PMC9240789 35753152

[43] Loughrey, M. B. et al. Identifying mismatch repair-deficient colon cancer: near-perfect concordance between immunohistochemistry and microsatellite instability testing in a large, population-based series. *Histopathology* **78**, 401–413 (2021). 32791559 10.1111/his.14233

[44] Harrell, F. E. *Regression Modeling Strategies: With Applications to Linear Models, Logistic Regression, and Survival Analysis*. (Springer, New York, NY, 10.1007/978-1-4757-3462-1 (2001).

[45] Hanley, J. A. & McNeil, B. J. The meaning and use of the area under a receiver operating characteristic (ROC) curve. *Radiology* **143**, 29–36 (1982). 7063747 10.1148/radiology.143.1.7063747

[46] Naing, L., Nordin, R. B., Abdul Rahman, H. & Naing, Y. T. Sample size calculation for prevalence studies using Scalex and ScalaR calculators. *BMC Med. Res. Methodol.* **22**, 209 (2022). 35907796 10.1186/s12874-022-01694-7 PMC9338613

[47] Arslan, S. et al. A systematic pan-cancer study on deep learning-based prediction of multi-omic biomarkers from routine pathology images. *Commun. Med.* **4**, 48 (2024). 38491101 10.1038/s43856-024-00471-5 PMC10942985

[48] Macenko, M. et al. A method for normalizing histology slides for quantitative analysis. in *2009 IEEE International Symposium on Biomedical Imaging: From Nano to Macro* 1107–1110 10.1109/ISBI.2009.5193250 (2009).

[49] Kather, J. N., Halama, N. & Marx, A. 100,000 histological images of human colorectal cancer and healthy tissue. Zenodo 10.5281/zenodo.1214456 (2018).

[50] Chen, X., Fan, H., Girshick, R. & He, K. Improved Baselines with Momentum Contrastive Learning. Preprint at 10.48550/arXiv.2003.04297 (2020).

