# Supplementary material for: H&E-based MSI/MMR testing with AI in colorectal cancer: a multi-centred blinded evaluation
Source: NPJ Digit Med. 2025 Dec 15;9:44. doi: 10.1038/s41746-025-02218-5 (PMC12800188; doi:10.1038/s41746-025-02218-5)
Supplement: Supplementary file 1 — Supplementary Materials [file 41746_2025_2218_MOESM1_ESM.docx]

## Supplementary Materials

|  | Cohort | | | | | | |
| --- | --- | --- | --- | --- | --- | --- | --- |
|  | L1-UK-CRC-SVS-1 -DEV  n (%) | N1-UK-CRC-SVS-1 -DEV  n (%) | I1-UK-CRC-CZI-1  -DEV  n (%) | T1-US-CRC-SVS-1  -DEV  n (%) | L1-UK-CRC-SVS-1 -BLIND  n (%) | W1-UK-CRC-CZI-1 -BLIND  n (%) | N1-UK-CRC-SVS-1 -BLIND  n (%) |
| Resections | 142  (100.00%) | 130  (100.00%) | 690  (82.63%) | 523  (94.40%) | 736 (100.00%) | 54 (100.00%) | 398 (100.00%) |
| Biopsies | 0 (0.00%) | 0 (0.00%) | 145 (17.37%) | 31 (5.60%) | 0 (0.00%) | 0 (0.00%) | 0 (0.00%) |
| Unknown | 0 | 0 | 176 | 0 | 55 | 0 | 0 |
| Total | 142 | 130 | 1011 | 554 | 791 | 54 | 398 |

**Supplementary Table S1 |** **Breakdown of biopsies and resections**. The numbers enclosed in parentheses are percentages calculated relative to the total number of patients in the cohorts (not including unknowns).

|  | | Cohort | | | | |
| --- | --- | --- | --- | --- | --- | --- |
|  |  | L1-UK-CRC-SVS-1-DEV  n (%) | I1-UK-CRC-CZI-1-DEV  n (%) | T1-US-CRC-SVS-1-DEV  n (%) | L1-UK- CRC-SVS-1-BLIND n (%) | W1-UK-CRC-CZI-1-BLIND  n (%) |
| Age (years) | n | 142 | 1011 | 554 | 791 | 54 |
|  | Unknown | 0 | 314 | 0 | 0 | 0 |
|  | Mean | 68.9 | 65.6 | 67.0 | 67.3 | 65.9 |
|  | STD | 12.8 | 12.8 | 12.3 | 12.2 | 11.6 |
|  | Median | 70.5 | 67.0 | 68.0 | 69 | 67.0 |
|  | Min | 32.0 | 19.0 | 31.0 | 23 | 40.0 |
|  | Max | 90.0 | 97.0 | 90.0 | 91 | 84.0 |
| Age Group n (%) | <50 | 11 (7.75%) | 72 (10.33%) | 54 (9.75%) | 67 (8.47%) | 6 (11.11%) |
|  | 50-65 | 41 (28.87%) | 233 (33.43%) | 156 (28.16%) | 235 (29.71%) | 16 (29.63%) |
|  | 65-75 | 33 (23.24%) | 200 (28.69%) | 180 (32.49%) | 250 (31.61%) | 16 (29.63%) |
|  | >75 | 57 (40.14%) | 192 (27.55%) | 161 (29.06%) | 239 (30.21%) | 16 (29.63%) |
| Sex n (%) | Male | 74 (52.11%) | 362 (51.94%) | 297 (53.80%) | 447 (56.51%) | 29 (53.70%) |
|  | Female | 68 (47.89%) | 335 (48.06%) | 255 (46.20%) | 344 (43.49%) | 25 (46.30%) |
|  | Unknown | 0 | 0 | 2 | 0 | 0 |

**Supplementary Table S2 | Study population demographics, highlighting age and sex distribution statistics among patients in each cohort.** Note that demographic data from the N1-UK-CRC-SVS-1-BLIND/-DEV cohorts and ethnicity information for the entire study were unavailable. The numbers enclosed in parentheses are percentages calculated relative to the total number of patients in each cohort (not including unknowns).

|  | | Cohort | | | | | | |
| --- | --- | --- | --- | --- | --- | --- | --- | --- |
|  |  | L1-UK-CRC-SVS-1 -DEV n (%) | N1-UK-CRC-SVS-1 -DEV n (%) | I1-UK-CRC-CZI-1-DEV n  (%) | T1-US-CRC-SVS-1-DEV n  (%) | L1-UK-CRC-SVS-1 -BLIND n (%) | W1-UK-CRC-CZI-1 -BLIND n (%) | N1-UK-CRC-SVS-1 -BLIND n (%) |
| Site of Tumour n (%) | Colon | N/A | N/A | 452  (64.85%) | 407  (73.86%) | 13  (1.65%) | 31 (57.41%) | 5  (1.26%) |
|  | Rectum | 22  (16.42%) | 1  (0.77%) | 245  (35.15%) | 78  (14.16%) | 262  (33.15%) | 23 (42.59%) | 1  (0.25%) |
|  | Two or more synchronous tumours in colorectum | N/A | N/A | N/A | N/A | 6  (0.76%) | N/A | 0  (0.00%) |
|  | Rectosigmoid | 1  (0.75%) | 1  (0.77%) | N/A | 66  (11.98%) | 4  (0.51%) | N/A | 5  (1.26%) |
|  | Transverse colon | 12  (8.96%) | 13  (10.00%) | N/A | N/A | 51  (6.46%) | N/A | 39  (9.80%) |
|  | Caecum | 32  (23.88%) | 28  (21.54%) | N/A | N/A | 104  (13.16%) | N/A | 111  (27.89%) |
|  | Ascending colon | 29  (21.64%) | 27  (20.77%) | N/A | N/A | 85  (10.76%) | N/A | 44  (11.06%) |
|  | Sigmoid colon | 29  (21.64%) | 38  (29.23%) | N/A | N/A | 172  (21.77%) | N/A | 114  (28.64%) |
|  | Splenic flexure | 1  (0.75%) | 8  (6.15%) | N/A | N/A | 28  (3.54%) | N/A | 24  (6.03%) |
|  | Hepatic flexure | 4  (2.99%) | 8  (6.15%) | N/A | N/A | 31  (3.92%) | N/A | 27  (6.78%) |
|  | Descending colon | 4  (2.99%) | 6  (4.62%) | N/A | N/A | 34  (4.30%) | N/A | 28  (7.04%) |
|  | Colorectal (specific location unknown) | 8 | N/A | 314 | 3 | 1 | N/A | N/A |
|  | Total | 142 | 130 | 1011 | 554 | 791 | 54 | 398 |

**Supplementary Table S3** **|** **Detailed tumour site**. The numbers enclosed in parentheses are percentages calculated relative to the total number of patients in the cohorts (not including unknowns).

|  | Cohort | | | | | | |
| --- | --- | --- | --- | --- | --- | --- | --- |
| Tumour morphological subtypes | L1-UK-CRC-SVS-1 -DEV  n (%) | N1-UK-CRC-SVS-1 -DEV  n (%) | I1-UK-CRC-CZI-1  -DEV  n (%) | T1-US-CRC-SVS-1  -DEV  n (%) | L1-UK-CRC-SVS-1 -BLIND n (%) | W1-UK-CRC-CZI-1 -BLIND n (%) | N1-UK-CRC-SVS-1 -BLIND n (%) |
| Adenocarcinoma | 104  (73.24%) | 109  (85.16%) | 932  (93.48%) | 338  (85.57%) | 678  (85.93%) | 51  (94.44%) | 316  (79.40%) |
| Mucinous adenocarcinoma | 29  (20.42%) | 18  (14.06%) | 64  (6.42%) | 57  (14.43%) | 101  (12.80%) | 3  (5.56%) | 77  (19.35%) |
| Signet ring adenocarcinoma | 4  (2.82%) | 1  (0.78%) | 1  (0.10%) | 0  (0.00%) | 7  (0.89%) | 0  (0.00%) | 3  (0.75%) |
| Micropapillary adenocarcinoma | 0  (0.00%) | 0  (0.00%) | 0  (0.00%) | 0  (0.00%) | 1  (0.13%) | 0  (0.00%) | 0  (0.00%) |
| Medullary carcinoma | 5  (3.52%) | 0  (0.00%) | 0  (0.00%) | 0  (0.00%) | 2  (0.25%) | 0  (0.00%) | 2  (0.50%) |
| Unknown | 0 | 2 | 14 | 159 | 2 | 0 | 0 |
| Total | 142 | 130 | 1011 | 554 | 791 | 54 | 398 |

**Supplementary Table S4 |** **Tumour morphological subtypes**. The numbers enclosed in parentheses are percentages calculated relative to the total number of patients in the cohorts (not including unknowns).

|  | L1-UK- CRC-SVS-1-DEV | | I1-UK-CRC-CZI-1-DEV | | L1-UK- CRC-SVS-1-BLIND | |
| --- | --- | --- | --- | --- | --- | --- |
| Histological grade | MSI-H/dMMR  n (%) | non-MSI-H/pMMR n (%) | MSI-H/dMMR n (%) | non-MSI-H/pMMR n (%) | MSI-H/dMMR  n (%) | non-MSI-H/pMMR n (%) |
| Well differentiated | 0 (0.00%) | 6 (5.13%) | 0 (0.00%) | 6 (3.90%) | 13 (14.13%) | 73 (10.55%) |
| Moderately differentiated | 10 (47.62%) | 90 (76.92%) | 8 (80.00%) | 133 (86.36%) | 52 (56.52%) | 560 (80.92%) |
| Poorly differentiated | 11 (52.38%) | 21 (17.95%) | 2 (20.00%) | 15 (9.74%) | 27 (29.35%) | 55 (7.95%) |
| Not recorded | 0 (0.00%) | 0 (0.00%) | 0 (0.00%) | 0 (0.00%) | 0 (0.00%) | 4 (0.58%) |
| Unknown | 2 | 2 | 125 | 722 | 1 | 6 |
| Total | 23 | 119 | 135 | 876 | 93 | 698 |

**Supplementary Table S5 |** **Breakdown of histological grading**. The distribution of microsatellite instability status (MSI-H/non-MSI-H) or mismatch repair deficiency status (dMMR/pMMR) is also provided. The numbers enclosed in parentheses are percentages calculated relative to the total number of patients (not including unknowns) in the cohorts. Tumour grading results that fell between categories, such as well/moderately differentiated, were grouped with the lower differentiation grade, in this case, moderate.

|  | L1-UK- CRC-SVS-1 -DEV | | N1-UK- CRC-SVS-1 -DEV | | I1-UK-CRC-CZI-1-DEV | | T1-US-CRC-SVS-1-DEV | |
| --- | --- | --- | --- | --- | --- | --- | --- | --- |
| Stage | MSI-H/ dMMR  n (%) | non-MSI-H/ pMMR  n (%) | MSI-H/ dMMR  n (%) | non-MSI-H/ pMMR  n (%) | MSI-H/ dMMR  n (%) | non-MSI-H/ pMMR  n (%) | MSI-H/ dMMR  n (%) | non-MSI-H/ pMMR  n (%) |
| I | 1  (4.35%) | 0  (0.00%) | 0  (0.00%) | 0  (0.00%) | 5  (7.58%) | 58  (11.91%) | 17  (22.37%) | 78  (17.11%) |
| II | 1  (4.35%) | 3  (2.54%) | 16  (59.26%) | 61  (59.22%) | 36  (54.55%) | 159  (32.65%) | 44  (57.89%) | 154  (33.77%) |
| III | 18  (78.26%) | 84  (71.19%) | 11  (40.74%) | 42  (40.78%) | 20  (30.30%) | 222  (45.59%) | 13  (17.11%) | 148  (32.46%) |
| IV | 3  (13.04%) | 31  (26.27%) | 0  (0.00%) | 0  (0.00%) | 5  (7.58%) | 48  (9.86%) | 2  (2.63%) | 76  (16.67%) |
| Unknown | 0 | 1 | 0 | 0 | 72 | 386 | 1 | 21 |
| Total | 23 | 119 | 27 | 103 | 138 | 873 | 77 | 477 |

**Supplementary Table S6 |** **Breakdown of cancer stage for the unblinded cohorts**. The distribution of microsatellite instability status (MSI-H/non-MSI-H) or mismatch repair deficiency status (dMMR/pMMR) is also provided. The numbers enclosed in parentheses are percentages calculated relative to the total number of patients (not including unknowns) in the cohorts.

|  | L1-UK- CRC-SVS-1 -BLIND | | W1-UK- CRC-CZI-1-BLIND | | N1-UK- CRC-SVS-1-BLIND | |
| --- | --- | --- | --- | --- | --- | --- |
| Stage | MSI-H/ dMMR  n (%) | non-MSI-H/ pMMR  n (%) | MSI-H/ dMMR  n (%) | non-MSI-H/ pMMR  n (%) | MSI-H/ dMMR  n (%) | non-MSI-H/ pMMR  n (%) |
| I | 14 (14.89%) | 143 (20.52%) | 1 (9.09%) | 4 (9.30%) | 0 (0.00%) | 0 (0.00%) |
| II | 58 (61.70%) | 255 (36.59%) | 5 (45.45%) | 22 (51.16%) | 47 (57.32%) | 189 (59.81%) |
| IIA / IIIB | 0 (0.00%) | 1 (0.14%) | 0 (0.00%) | 0 (0.00%) | 0 (0.00%) | 0 (0.00%) |
| III | 20 (21.28%) | 218 (31.28%) | 5 (45.45%) | 15 (34.88%) | 35 (42.68%) | 127 (40.19%) |
| IV | 2 (2.13%) | 80 (11.48%) | 0 (0.00%) | 2 (4.65%) | 0 (0.00%) | 0 (0.00%) |
| Unknown | 0 | 0 | 0 | 0 | 0 | 0 |
| Total | 94 | 697 | 11 | 43 | 82 | 316 |

**Supplementary Table S7 |** **Breakdown of cancer stage for the blinded cohorts**. The distribution of microsatellite instability status (MSI-H/non-MSI-H) or mismatch repair deficiency status (dMMR/pMMR) is also provided. The numbers enclosed in parentheses are percentages calculated relative to the total number of patients (not including unknowns) in the cohorts.

| Model backbone | Sample size n (*Unstable %*) | C- statistic | Overall Percent Agreement %  (CI) | Positive Percent Agreement %  (CI) | Negative Percent Agreement % (CI) | Test Replacement Rate % |
| --- | --- | --- | --- | --- | --- | --- |
| ImageNet | 551 (19.78%) | 0.93 | 91.48 (88.54-93.87) | 87.78  (79.18-93.74) | 92.39 (89.19-94.88) | 83.12 |
| SSL | 551 (19.78%) | 0.96 | 95.25 (92.89-97.00) | 95.51 (88.89-98.76) | 95.19 (92.50-97.12) | 84.03 |

**Supplementary Table S8 |** **Five-fold cross-validation results for models using an SSL-pretrained backbone vs. ImageNet, applied to the L1-UK-CRC-SVS-1-DEV cohort.** The c-statistic represents the aggregated predictions from all five models (one per fold), covering the entire dataset.

|  | True Status | |
| --- | --- | --- |
| Predicted Status | Positive | Negative |
| Positive | TP | FP |
| Negative | FN | TN |
| Indeterminate | IP | IN |

**Supplementary Table S9 | Confusion matrix used for calculating evaluation metrics**. TP: True positive. FP: False positive. FN: False negative. TN: True negative. IP: Indeterminate positive, i.e., positive cases for which PPC returned an Indeterminate result. IN: Indeterminate negative, i.e., negative cases where PPC returned an Indeterminate result.

| Performance Metric | Definition |
| --- | --- |
| Overall Percent Agreement (OPA) / Concordance (Accuracy) | $\frac{TP+TN}{TP+TN+FP+FN}$ |
| Positive Percent Agreement (PPA) / Sensitivity | $\frac{TP}{TP + FN}$ |
| Negative Percent Agreement (NPA) / Specificity | $\frac{TN}{TN + FP}$ |
| Positive Predictive Value (PPV) / Precision | $\frac{TP}{TP + FP}$ |
| Negative Predictive Value (NPV) | $\frac{TN}{TN + FN}$ |
| False Negative Rate (FNR) | $\frac{FN}{TP + FN}$ |
| False Positive Rate (FPR) | $\frac{FP}{TN + FP}$ |
| Test Replacement Rate (TRR) | $\frac{TP+TN+FP+FN}{TP+TN+FP+FN+IP+IN}$ |

**Supplementary Table S10 | Definitions of the performance metrics used for the evaluation of PANProfiler Colorectal (PPC).** Standard diagnostic measures used to measure PPC performance. TP: True positive. FN: False negative. TN: True negative. FP: False positive. IP: Indeterminate positive, i.e. positive cases where PPC returned an Indeterminate result. IN: Indeterminate negative, i.e., negative cases where PPC returned an Indeterminate result. Overall, positive, and negative percent agreement are computed by comparing against standard tests, and correspond to accuracy (or concordance), sensitivity and specificity, respectively.


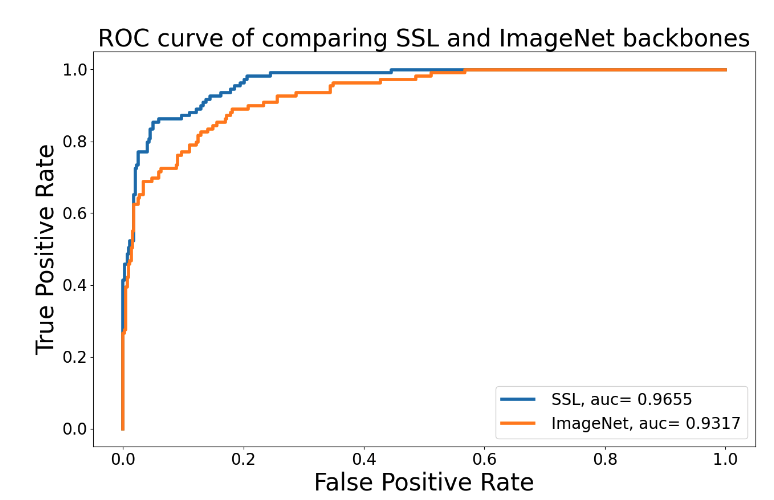


**Supplementary Figure S1 | Receiver operating characteristic (ROC) curve comparing SSL and ImageNet backbones applied to the L1-UK-CRC-SVS-1-DEV cohort.** Test AUC is the result of the aggregation of all predictions across the 5 models, which includes the entire dataset. AUC is also known as the C-statistic.


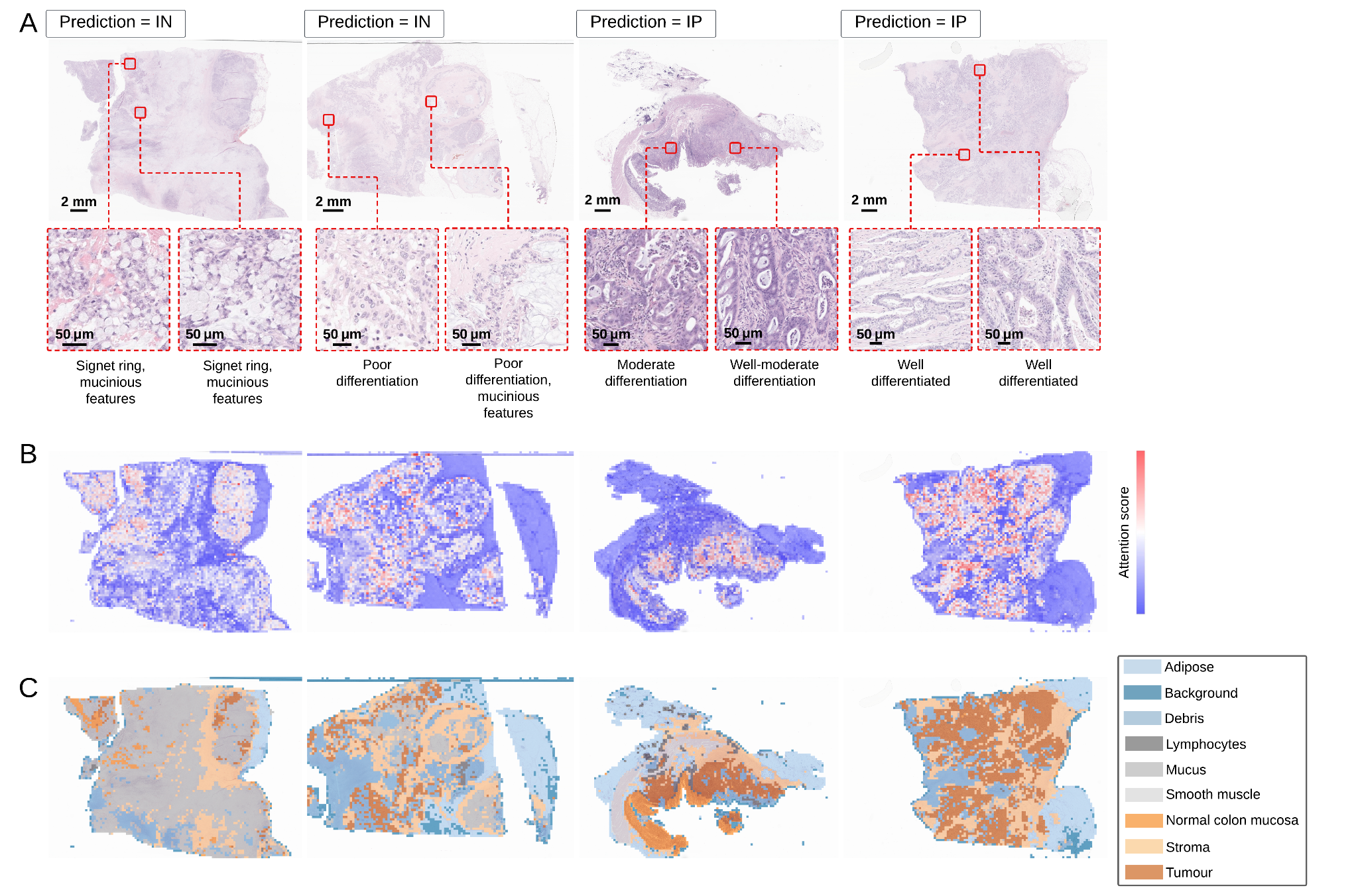


**Supplementary Figure S2 | Explainability and interpretability visualisations** for images that were classified as Indeterminate by the model, where two Indeterminate Negative (IN, non-MSI-H/pMMR) and two Indeterminate Positive (IP, MSI-H/dMMR) cases from the L1-UK-CRC-SVS-1-BLIND cohort are shown. **A**: Original WSI and two regions of interest (ROIs) beneath it (selected from different regions indicated by the red boxes). **B**: Attention scores with darker hues of red indicating high attention and blue indicating low attention. **C**: Annotated regions based on a classification of the tissue, including adipose, background, debris, lymphocytes, mucus, smooth muscle, normal colon mucosa, stroma, and tumour.

**
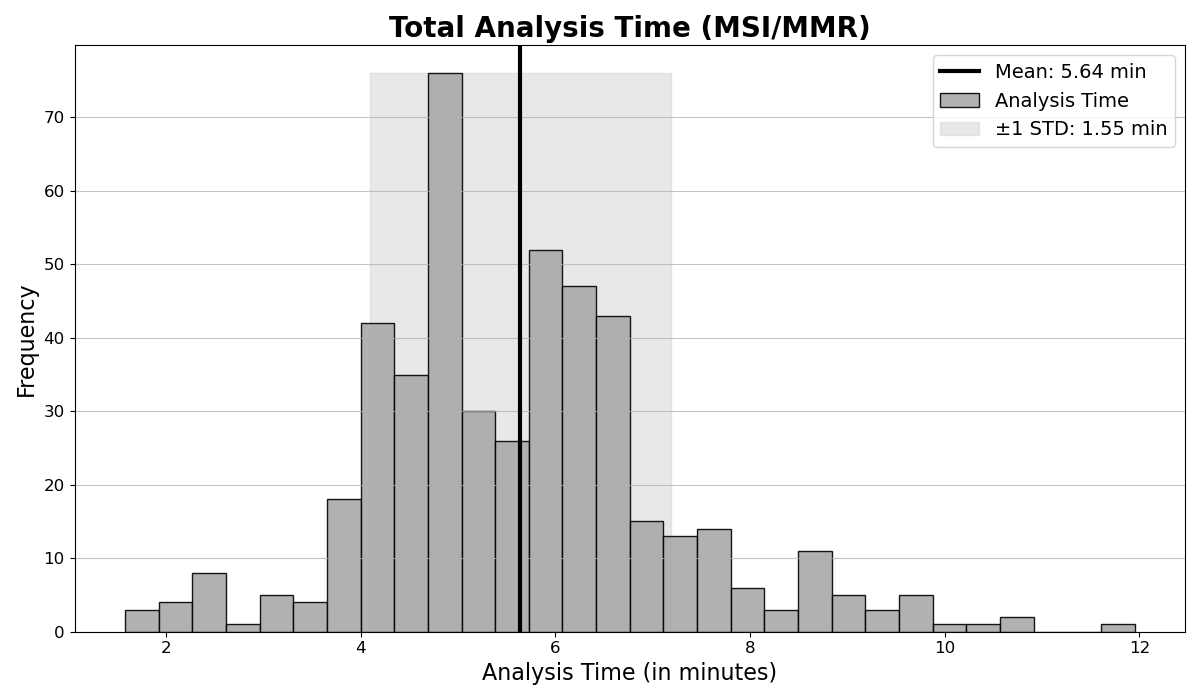
**

**Supplementary Figure S3 | Distribution of analysis times for MSI/MMR test cases**. The histogram represents the total prediction total analysis time (in minutes) across multiple test cases. The mean analysis time is indicated by a solid black line, and the shaded region represents one standard deviation from the mean. These visualisations highlight the variability and central tendencies in prediction times, providing insights into the computational efficiency for each biomarker test.
